# Where did the vessels go? An analysis of the EU fishing fleet gravitation between home ports, fishing grounds, landing ports and markets

**DOI:** 10.1371/journal.pone.0230494

**Published:** 2020-05-21

**Authors:** Steven Holmes, Fabrizio Natale, Maurizio Gibin, Jordi Guillen, Alfredo Alessandrini, Michele Vespe, Giacomo Chato Osio

**Affiliations:** European Commission, Joint Research Centre (JRC), Ispra, Italy; Aristotle University of Thessaloniki, GREECE

## Abstract

The mobile nature of fishing activity entails dynamic spatial relations and dependencies between coastal communities and fishing grounds drawn by the movement of fishing vessels. Analysing these spatial relations is essential to allocate the socio-economic impact of the fishing activity into the relevant coastal communities. In addition, such spatial information gives the possibility, on the one hand, to assess the impacts from fisheries on the marine environment and, on the other, to manage competing uses of the sea space between different activities. In this paper, we use AIS data, which is individual vessels’ positioning data, to examine the activity of the EU large-scale fishing fleets, their home ports, high intensity fishing areas (i.e., main fishing grounds), main ports and coastal communities involved.

## Introduction

The mobile nature of fishing activity entails dynamic spatial relations and dependencies between coastal communities and fish species in a given area (fishing grounds). Analysing these dynamics is essential in order to allocate the economic effects, usually expressed in terms of gross value added (GVA) and employment opportunities, to the relevant coastal communities. In addition, such information gives the possibility, to assess the impacts from fisheries on the marine environment and to manage the use of the sea space between the competing interests of fisheries, other maritime economic sectors and environmental protection objectives. Competing interests among the different sectors are increasingly regulated through spatial measures such as Maritime Spatial Plans (MSPs), Territorial Use Rights for Fishing (TURF) or Marine Protected Areas (MPAs) [1]. Such measures require an integrated approach that considers fisheries in relation to other economic sectors and activities. In the case of the European Union (EU), this means managing fisheries not only under the Common Fishery Policy (CFP) but also in the broader context of the Integrated Maritime Policy (IMP) and the Marine Strategy Framework Directive (MSFD).

Most studies incorporating socio-economic analysis to the spatial dimension of fisheries have investigated food security in fisheries at national level [2–6], but few of them have investigated the socio-economic impact of fisheries in the local communities.

The socio-economic performance of the fisheries sector for the entire EU is analysed yearly at fleet segment level by the Scientific, Technical and Economic Committee for Fisheries (STECF)’s Annual Economic Report of the EU Fishing Fleet [7,8]. Given the high level of aggregation of the disseminated output, it is not possible to appreciate the relevance of fishing activities for specific coastal communities. A disaggregation of GVA and employment figures at the level of coastal communities is provided in Natale et al. [9]. In the paper, the authors assumed that the homeport of the fishing vessels declared in the EU fleet register can be considered as the main place of gravity for the allocation of socio-economic indicators related to the fishing activity. This assumption is robust for small fishing vessels, as they have a limited operational range, but larger vessels are expected to operate at greater distances, exploiting alternative fishing opportunities and markets [10].

The movement of large-scale fleets to distant fishing grounds and fish markets makes it difficult to identify a single location to attribute the economic effects of their activity. The study of the complex spatial dynamics emerging from the movement of fishing vessels represent a particularly interesting field of study for economic geography compared to other industrial and agricultural sectors, for which the economic activity is fixed.

Traditionally, most of the models used to understand the distribution and the localisation of markets and economic activities derive from Reilly's Law of retail gravity [11], which draws from Newton's law of universal gravitation and has been widely used to identify trade areas between cities by considering the attraction effect of the ‘mass’, expressed as the population of a town centre. After Reilly’s Law, more complex models were developed to account for additional measures of the attraction of a town centre. In particular Wilson [12], applied the concept of entropy to flows and developed a general spatial interaction model, of which the gravity model can be considered a special case.

The application of gravity and spatial interaction models to fisheries is facilitated by the increasing availability of detailed vessel positioning data from the Vessel Monitoring System (VMS) and the Automatic Identification System (AIS), which improves the spatial-temporal resolution of fishing activity data compared to classical logbooks.

In the EU, aggregated data on effort and landings are transmitted by the Member States in response to data calls by STECF, by the International Council for the Exploration of the Sea (ICES), by the General Fisheries Commission for the Mediterranean (GFCM) and other end-users. The data have a coarse spatial resolution, namely Food and Agriculture Organization (FAO) fishing areas, GFCM Geographical Subareas (GSAs) or ICES rectangles (1° longitude by 0.5° latitude), and a temporal resolution by year, quarter or month. Primary data for individual vessels from logbooks have a spatial resolution of ICES rectangles or fishing areas in the Mediterranean, a temporal approximation of 24 hours and are only available to national control authorities or fisheries research institutes. AIS and VMS provide exact positions of individual vessels at temporal intervals ranging from 30 minutes to 2 hours, for VMS, to a few minutes or seconds, in the case of AIS. In fisheries research, there are now many applications leveraging the spatial-temporal resolutions of AIS and VMS data to complement the aggregated figures derived from logbooks [13–15]. High resolution fishing activity data gives the opportunity to study the economics of fisheries using retail and data science modelling methods, which examine the social and economic behaviour of individual agents rather than of aggregated economic segments.

In our model, we considered each vessel as an agent whose behaviour is described by the distribution of AIS messages over time and space. The movements of the vessels define relations and dependencies between the coastal communities of origin, the areas mostly fished and other ports visited. High Intensity Fishing Areas (HIFA) are determined by setting a percentile on the cumulative density distribution of the points classified as fishing.

The first objective was to verify if the port most commonly visited by a vessel coincided with the home port declared in the EU fleet register and, if this was not the case, to look for a reliable geographical centre of gravity for each vessel. The correct identification of a fishing vessel’s centre of gravity improves the allocation of the economic effects of the fishing activity to port level. Once the ports of gravity were identified and the interactions with other ports mapped, it was tested whether the dynamics between ports may be explained by the presence of fish markets. In particular, we would expect the presence of large fish markets, with larger demand, efficient infrastructures and favourable selling conditions, to explain the preference of large-scale fishing vessels having their homeport in other ports or countries.

The second objective was to define the HIFA and introduce a spatially explicit methodology to explore how individual vessels and coastal communities interact. While the AIS data would give the possibility to define HIFA and analyse “who is fishing where” at the level of single vessel, we present our results at a relatively high level of aggregation of group of vessels in each port. This choice is determined by the scale of the analysis, covering the entire EU, by the focus of the analysis, which is on economic relations between fishing coastal communities and HIFA, and by confidentiality requirements, which prevent showing information and data that can be traced back to individual persons or enterprises [16].

The analysis is structured in four main parts. First, the HIFA were established. Secondly, port-to-HIFA and port-to-port relations were calculated by counting the number of (temporally standardised) AIS messages falling inside the boundaries of the HIFA and in the proximity of the ports. Third, these relations were analysed using a standard analytical tool from the network analysis literature to calculate the nodes’ centrality and the presence of clusters. Finally, official statistics data on employment in fisheries and Gross Value Added were allocated to the homeport on the “land-side” and to the HIFA on the “sea-side”.

## Methods

The raw data considered in the study was obtained from the JRC Blue Hub platform (https://bluehub.jrc.ec.europa.eu) and comprised almost 2.5 billion vessel positioning AIS messages. Vessels equipped with Class A transponders, broadcast messages containing two main types of information, dynamic information: transmitted every 2 to 10 seconds while the vessel is underway and every 6 minutes while anchored; static & voyage related Information: provided by the vessel's crew and transmitted every 6 minutes regardless of the vessel's movement status. Dynamic information includes: Maritime Mobile Service Identity number (MMSI)—a unique identification number for each vessel station (the vessel's flag can also be deducted from it); AIS Navigational Status; rate of turn (ROT); speed over ground (SOG); Latitude/Longitude; course over ground (COG); Heading; Bearing and UTC (Coordinated Universal Time).

The ‘static’ part of AIS messages contains the International Maritime Organisation number (IMO); Call Sign (international radio call sign assigned by the country of registry); vessel name; the type of ship; dimensions; location of the positioning system's antenna on board the vessel; type of positioning system (GPS, DGPS, Loran-C); draught; destination and estimated time of arrival.

Because most of the static information is input by the vessel’s crew it can be prone to errors. It is not possible however, to discard all static information as some of it is used to join additional information.

Although the AIS was originally conceived for safety and collision avoidance, it has also been used for data-driven knowledge extraction related to maritime uses such as shipping, fishing, and human related activities at sea [17,18]. In the EU, AIS is mandatory for vessels above 15 meters length over all (LOA), such that fishing vessels above 15 meters LOA was our definition of ‘large scale’ fishing fleet.

From the AIS data set approximately 123.5 million messages were extracted and resampled at 5 minutes intervals. The data related to 6073 EU fishing vessels operating in FAO areas 27 (North East Atlantic), area 37 (Mediterranean and Black Sea) and area 34 (Central Eastern Atlantic), in the period between September 2014 and September 2015. This represented 68% of the fishing vessels above 15 meters LOA in the EU fishing fleet register. Gear attribution was obtained from a merge with the EU fishing fleet register. The AIS ‘fleet’ was comprised 70% of trawlers, 12% of purse seiners and 18% of other gears.

The level of coverage of the AIS data set was different across countries. For Belgium, Denmark and Sweden, more than 80% of the large-scale fishing fleet was represented, whereas for Romania, Cyprus and Slovenia less than 50% of the fleet above 15 meters length was represented. The total number of days by vessel for which data was available was approximately 1.18 million. This represents 70% of the days at sea notified under the EU Data Collection Framework (DCF) for vessels above 12 meters of length [7], a conservative estimate of coverage since official figures of effort include vessels between 12 and 15 meters LOA.

**Fig 1:**
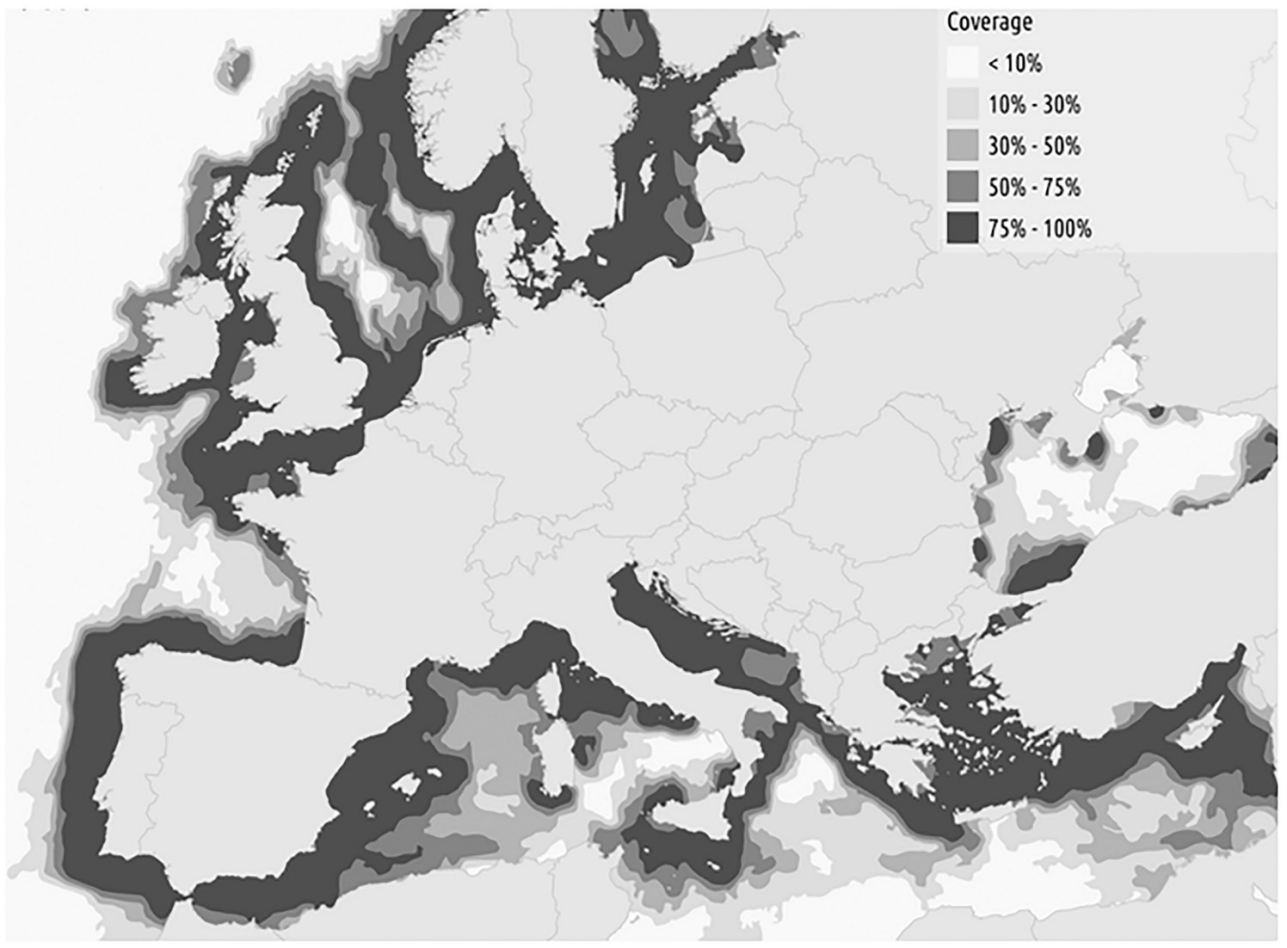
Assessment of the AIS data coverage. The map displays the median of normalised values after the assessment of coverage was performed monthly.

The spatial coverage of AIS is dependent on the propagation of the radio signal, on the presence of ground or satellite based receiving stations and ultimately on the operational range of the vessel. It is therefore necessary to compute and consider maps of data coverage when attempting to draw conclusions from AIS data. Coverage analysis was carried out using merchant vessel routes extracted from the Long Range Identification and Tracking (LRIT) system. The LRIT data was used to generate a “transition matrix”, which provided the basis of a path-finding algorithm to generate the “most likely” vessel trajectories between two geographical coordinates. Then for each data cell the ratio between received and expected tracks of vessels travelling at normal cruising speed was calculated [18]. The resulting coverage map is shown in Fig 1.

Effort maps using the same AIS data source have been compared with the maps of fishing effort derived from VMS data produced by the 2016 ICES Working Group on Spatial Fisheries Data [15], additional comparisons were carried out by Shepperson [19] and Ferrà et al. [20]. The main conclusion from the comparisons was that AIS tends to underestimate the total fishing effort and to overestimate the share of trawlers, due to the gear information attribution from the EU fleet register.

For the definition of HIFA, firstly AIS messages were classified to either fishing or steaming using an unsupervised classification method of individual vessels’ speed profiles. The method relies on the idea that the majority of fishing vessels with towed gears operate at lower speed when fishing compared to when steaming. The speed frequencies show a typical bimodal distribution with the highest number of messages concentrated on the first mode. By fitting a multimodal distribution on the speed frequencies it is possible to identify the thresholds for the classification of messages as fishing or not [21,22]. Methods based on speed, or on a combination between speed and direction [23–25], proved to be sufficiently robust for vessels using towed gears (e.g. dredges, otter, beam and mid water trawls), which represent more than 90% of the vessels above 15 meters overall length in the EU fleet. For other gear types like purse seiners [26], the development of methods for the classification of fishing behaviour is still an open field of research.

Having classified the messages into fishing or steaming, a kernel density on the fishing messages raster was computed at a resolution of 1 by 1 km. On the resulting smoothed density map HIFA were defined in each FAO area as polygons including cells with a high frequency of messages. For the purpose of this analysis, through expert judgement and experience from previous research, we set the threshold to 75% of the cumulative density distribution. Small polygons of less than five square kilometres and holes within the HIFA boundaries were deleted.

A relation between a port and a HIFA was established on the basis of the number of messages for individual vessels from that port falling in the HIFA. On the resulting network a clustering analysis based on random walks was used to merge several ports and HIFA based on the most frequent interactions (i.e., the highest number of weights).

To calculate dependencies, individual fishing vessels and coastal communities were related to the HIFA and ports by summing, respectively, the number of messages classified as fishing falling inside the HIFA boundaries and the non transit messages in a buffer of 0.01 degrees radius around each port. The buffer around the ports was used to capture incoming and outgoing tracks and within port manoeuvres to compensate for the inaccuracies in geocoding with respect to the exact location of mooring areas. The port with the highest number of messages was considered as the centre of gravity or “real” homeport of the vessels and this information was compared to the declared homeport in the EU fleet register.

From the spatial distribution of individual fishing vessel movements two directed and weighted networks were created: one for the port-to-port relations and one for the port-to-HIFA relations. In the port-to-port network a directed edge was established between the homeport and the other ports visited by individual vessels. The weight of the directed edge was calculated as the total number of messages in the target port by vessels from a given home port. Similarly, for the port-to-HIFA relations, the edges were calculated as the number of messages in a given target HIFA by vessels coming from each home port. In both cases, the home port was defined by the newly established main centre of gravity rather than that declared in the fleet register.

Considering both ports and HIFA as network nodes we computed the nodes’ centrality as the total weights of the incoming edges [27]. In the case of the port-to-port network the centrality calculation was performed after excluding self-edges (landings back to the home port) and cases when vessels landed in ports outside of the study area. The calculation therefore represents the attraction of the ports to vessels having their homeport elsewhere.

The clustering of nodes was performed using the walk-trap community algorithm, which is a hierarchical network clustering approach based on random walks [28]. The algorithm performs short random walks on the network following the existing edges. The right number of clusters is based on the maximisation of a modularity score, calculated as the fraction of edges within the clusters minus the fraction in a random network.

The clustering gave a simplified representation of groups of interconnected fishing areas and ports, filtering out less frequent movements of vessels. For each cluster we estimated the number of employed persons and GVA through technical coefficients for the number of persons per vessel and labour productivity by length class, country and year obtained from DCF data.

To test if the attraction effect of some ports is associated with the presence of fish markets we compared the centrality measures in the port-to-port network with the average volume of sales (*weight × mean price*) in fish markets in the period 2011–2014. Data on fish sales volume and value was obtained from the European Market Observatory for Fisheries and Aquaculture (EUMOFA). The markets were geocoded and allocated to ports by matching the strings of the names and in case of non-successful matches by a proximity analysis. The dataset of geo referenced markets and ports covered around 1274 first sale fish markets in 13 countries, with ports linked to multiple markets. For the comparison to port centrality, markets were excluded if data was missing in more than one year used to calculate the average sales volume. Markets associated to the same port had their sales volumes summed. The coverage of the markets data was assessed by comparing the sum of sales across ports within a country in the period 2014 with the total value of landings into that country reported under the DCF [7].

## Results

**Fig 2:**
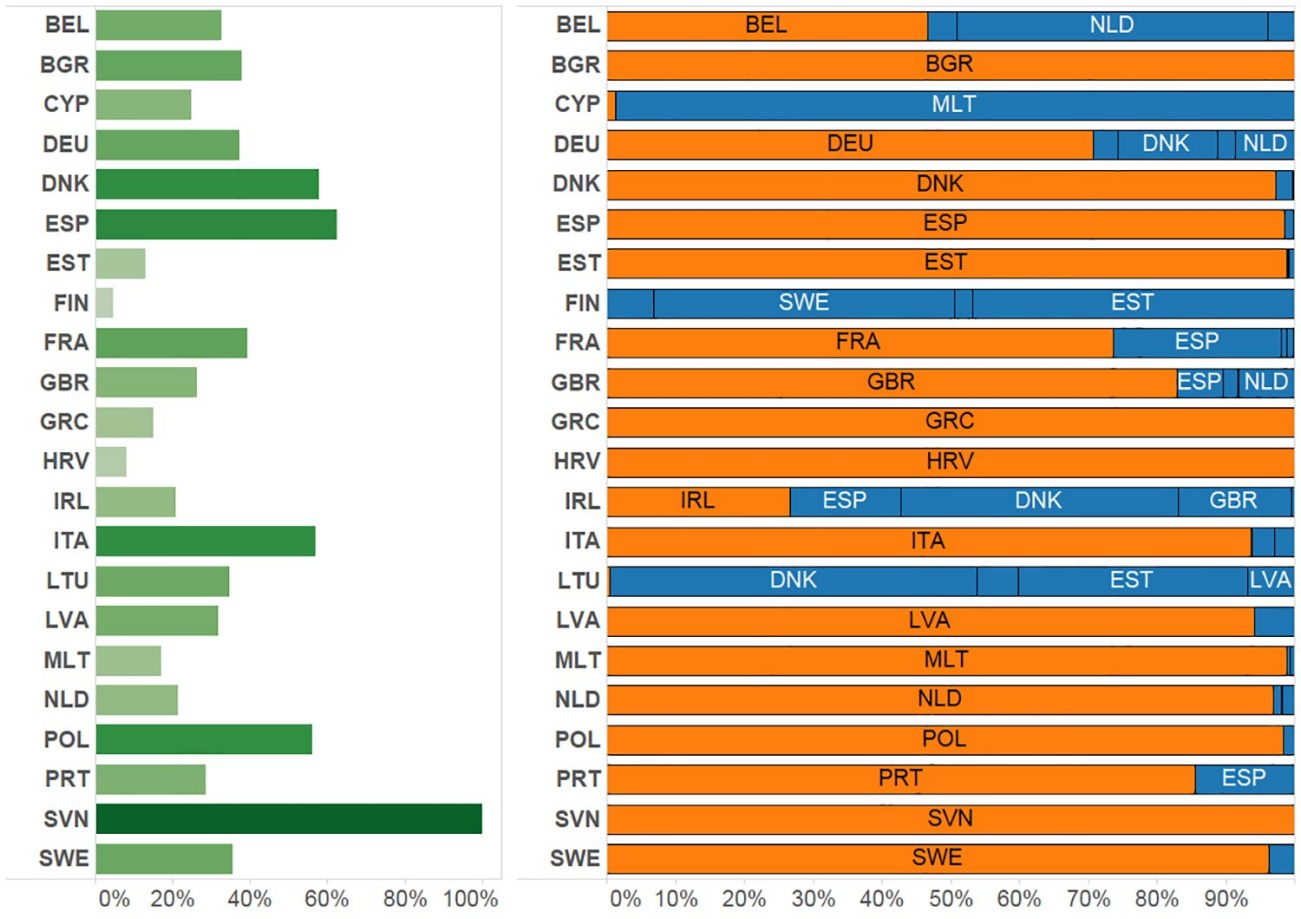
Gravity between ports. Left: percentage of vessels in each country for which the declared port in the fleet register coincides with the centre of gravity determined based on the number of AIS messages. Right: share of messages in ports of country of registration and ports of other countries. BEL: Belgium; BGR: Bulgaria; CYP: Cyprus; DEU: Germany; DNK: Denmark; ESP: Spain; EST: Estonia; FIN: Finland; FRA: France; GBR: UK; GRC: Greece; HRV: Croatia; IRL: Ireland; ITA: Italy; LTU: Lithuania; LVA: Latvia; MLT: Malta; NLD: Netherlands; POL: Poland; PRT: Portugal; SVN: Slovenia; SWE: Sweden.

The left side of Fig 2 shows the percentage of vessels for which the port declared in the fleet register coincides with the one identified through highest density of AIS messages. Overall there is a correspondence for 43% of the vessels of the EU fleet. Values below 20% were found in the case of the fleets of Finland (5%), Croatia (8%), Ireland (13%), Estonia (13%), Greece (15%) and Malta (17%). The right side of Fig 2 presents the number of messages from vessels landing at ports within the home country compared to ports in other countries. These results indicate how most of the activity of the fleets of Cyprus, Finland and Lithuania is centred in ports of other countries while fleets from Greece, Malta, Bulgaria, Slovenia, Poland and Croatia tend to gravitate almost exclusively around ports in their own country.

The cluster analysis of the port-to-port network identified 124 clusters. Of these, 23 clusters included more than 5 ports. The largest cluster in terms of number of vessels is located on the west coast of Spain and includes 387 vessels from 54 Spanish ports and 12 Portuguese vessels. The most central port in this cluster is the port of Ondarroa. The second largest cluster is centred in Denmark and includes vessels from Germany, Lithuania, Latvia, Poland and Sweden. The most central port in this cluster is the Danish port of Hirtsalhal.

**Fig 3:**
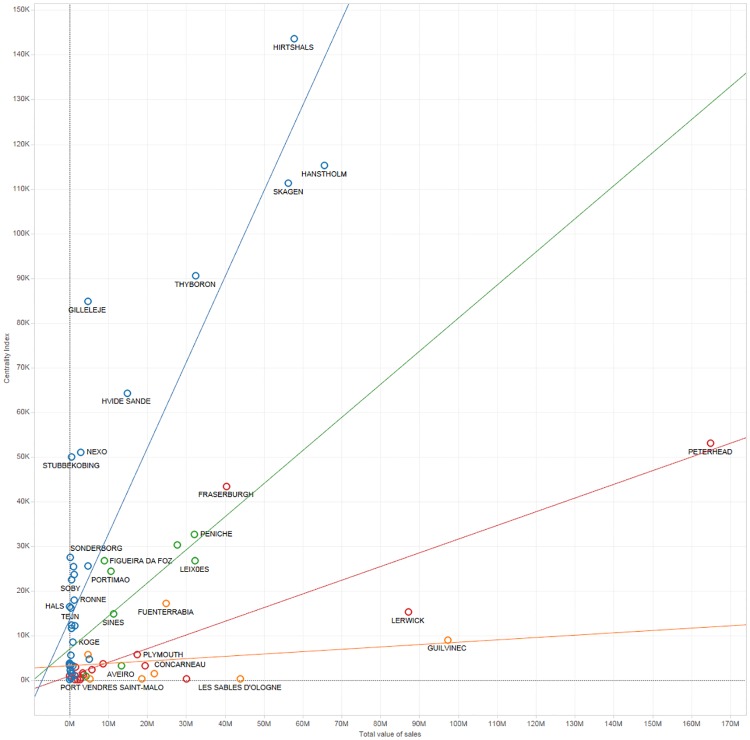
Relation between the centrality score of ports and the average annual value of fish sales in fish markets in the same location. Lines show simple linear regressions to data points from individual countries, blue: Denmark; green: Portugal; red: UK; orange: France.

The scatter plot in Fig 3 shows the relation between the centrality values of the ports and the average value of fish at first sale in the period 2011–2014 for markets located in the same localities. The coverage of market data was acceptable only for Ireland (84%), Belgium (83%), Latvia (74%), Denmark (73%), UK (73%), France (57%), Netherlands (55%) and Portugal (55%) and poor for Germany (7%), Italy (6%), Sweden (4%), Greece (2%) and Lithuania (1%). Fig 3 supports the idea of correlation between centrality and sales value but with very different relationships between different countries, as illustrated by the simple linear regressions to the data for Denmark, Portugal, the UK and France (other countries with reasonable data coverage were considered to have too few ports for meaningful results).

**Fig 4:**
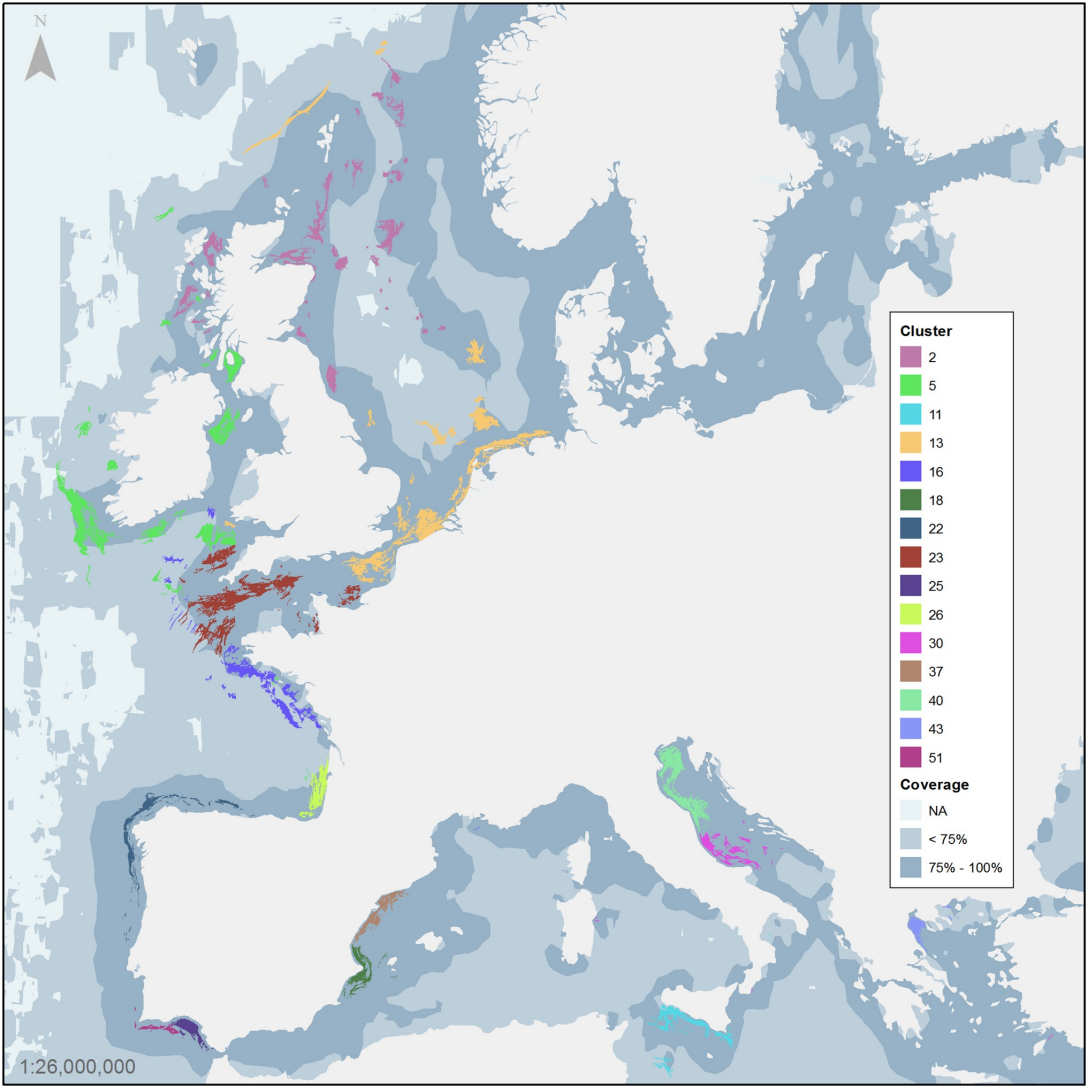
Ports- fishing ground clusters. The map shows the ports and HIFA belonging to the top 15 clusters in terms of employment (see also Table 1). Each cluster is represented by a separate colour.

The clustering of the port-to-HIFA network identified a total of 137 clusters. Fig 4 presents a map of the top 15 clusters in terms of total employment while Table 1 provides descriptive statistics on number of ports, number of vessels, number of HIFA, total area, employment and GVA for these clusters.

**Table 1:** Descriptive statistics for the top 15 port–HIFA clusters on the basis of total employment.

Cluster ID	Ports (N)	Vessels (N)	GVA (Million Euro)	Employment (N)	HIFA
5	23	779	332	5229	31
22	24	507	167	4902	37
13	54	888	299	3609	44
40	16	892	73	3030	13
26	19	274	124	2720	4
37	12	342	59	2385	10
25	10	346	49	2216	12
30	14	500	46	1818	17
2	15	290	115	1800	40
23	11	305	95	1480	33
16	9	305	115	1479	26
18	11	196	33	1405	12
11	11	361	35	1306	12
24	24	232	35	1032	11
31	27	363	111	1020	31

The main cluster in terms of employment is in FAO Area 27 along the Atlantic coast of Ireland and the Irish Sea. In this cluster, there are 23 ports, 31 HIFA and 779 vessels above 15 m of length, which generate an employment of 5229 persons and annual GVA of 332 million Euros corresponding to around 10% of the total GVA by EU fisheries in 2013. The second largest cluster is in FAO Area 37 along the coast of Catalonia, Valencia and Murcia regions (Spain); it includes 24 ports and 37 HIFA and has an employment of 4902 persons and a GVA of 167.4 million Euros. The third largest cluster in terms of fisheries employment (3609 persons) and largest in terms of HIFA area comprises 54 ports and 44 HIFA along the coast of the Netherlands, Belgium, the Strait of Dover and in the North Sea. The clustering of the ports and HIFA represent aggregates of the more intensive spatial interactions.

## Discussion

In this paper we examined the activity of the EU large-scale fishing fleets (vessels > 15 m LOA) using individual vessels’ positioning data to identify the homeport and define spatial relations between homeports, HIFA and other ports. The results showed that the large-scale fleets tend to spread their presence over several ports rather than gravitating exclusively to one port.

The comparison between the homeport declared in the fleet register and that emerging as centre of gravity from the analysis of AIS data showed a mixed picture across countries. The low correspondence for some countries indicates the need to use fishing activity data to find the correct centre of gravity so that spatially explicit fisheries economics data at the level of coastal communities can be allocated correctly. Frequent interactions with ports different from the homeport point to the need to redistribute the economic effects of fishing across sometimes distant geographic areas. This aspect, which is specific to the fishing sector and linked to the mobile nature of its production unit, is particularly evident in the case of vessels well over 15 m LOA (including high seas fleets) with larger operational ranges. This geographical separation is also applicable to employment, since often the crew is recruited close to the HIFA, and to the downstream effects on the processing industry and commercialisation of fish, since the fish is sold where market conditions are more favourable.

The flexible spatial behaviour of large vessels, that causes them often to gravitate towards ports in distant areas or countries rather than towards the declared home port, can intuitively be explained by better prices and market opportunities. In our analysis we empirically observe that high values of centrality in the port-port network are associated with the presence of large fish markets. The historic development of European markets is outside the scope of this study but we assume a unidirectional nature to this relation (the current attractiveness of ports to vessels based elsewhere depends on the size of first sale markets at or close to the port) based on two caveats: firstly that the centrality only considers vessels from other ports and secondly that the volume of sales is averaged over 2011–2014. There was also considerable difference in coverage of market data between countries. These differences can be explained by the lack of data provision agreements between EUMOFA and the private national organisations supplying price and market data in some countries.

Understanding the spatial relations on the “sea-side” between ports and HIFA gives the possibility to assess in a relatively straightforward way the impact on fisheries of different management measures defined for a given sea area. Impacts from a wide range of management measures such as establishing MPAs or closed areas (e.g., where fishing is limited or restricted), MSPs (e.g. changes of uses of certain areas, such as dedicating them for offshore wind energy), changes in the fish quotas for a species in a given area, changes in the regulation on EEZs (e.g. Brexit) and changes in the management and allocation of fishing licences or property rights (e.g. TURFs). By knowing the amount of fishing activity of a fishing community in a given HIFA it is possible to derive an estimate of the employment and GVA that would be affected in each fishing community, region or country.

The main issue in using AIS compared to VMS and logbooks for fisheries research is linked to its less systematic coverage that derives from AIS being an open system not specifically designed to monitor and control fisheries. The assessment of the coverage of the AIS is a complex issue, which is still open to further methodological research in the field of spatial statistics, signal processing and radio propagation. Radio propagation is usually limited to line-of-sight between the transmitter (AIS transponder on board the ship) and the receiver (land base station feeding the terrestrial network). In VHF frequencies, the signal propagation depends on many factors, including local atmospheric conditions, and may vary significantly between different months and even days of the year. According to the AIS data coverage obtained in this study, as shown in Fig 4, areas can be classified as having: a) complete or near complete AIS coverage, and consequently AIS data can be considered sufficient on its own, b) good AIS coverage, which is considered sufficient to define HIFA but, data can be supplemented with VMS data and c) an absence of coverage.

The variation in AIS coverage can produce anomalous results. Figs 1 and 4 show Ireland with a significant proportion of coastline with an AIS coverage of < 75% but very high coverage in most other coastal areas. Over the period of the AIS data studied, Irish registered vessels landed 94% of trips in Ireland and only 0.3% in Denmark, a result not reflected in Fig 2. Any intended study using the methodology would need to consider consistency of AIS coverage for the fleet(s) studied before drawing inferences.

Given the level of AIS coverage of the dataset used in this paper, we conclude that AIS does not provide by itself a reliable estimation of fishing effort in certain regions. In addition, a more thorough analysis is needed to compare fishing effort estimates between AIS, VMS and the DCF aggregated data. However, in terms of the analysis of the spatial and temporal distribution of fishing activity it has been shown that the AIS data used covers a large part of the EU fishing fleet in terms of number of vessels and number of days at sea and that the AIS data provides a similar spatial distribution of effort to VMS data (ICES, 2016).

AIS has the advantages of being openly accessible and more detailed in terms of frequency of messages which avoids the need for track reconstruction procedures normally necessary with VMS. Although not available to this study, increasing coverage of satellite received AIS signals is progressively eliminating AIS blind spots [29].

In our analysis all gears and métiers were aggregated when defining relations between vessels and HIFA. This is justified by the broad scope of the analysis and the focus on regional economics rather than bio economic modelling. More detailed gear specific representations of fishing ground would need to be considered to capture specific gear-stock relations or to assess the impacts on the seabed floor of the different gears.

Another issue in defining the HIFA is related to the threshold used to identify “high” intensity. Analyses of different methods for the definition of fishing grounds on the basis of VMS data showed how the full extent of areas fished is normally composed of relatively small high intensity core areas and relatively large margins with little activity [30]. The exclusion of these margins and the adoption of a common definition of fishing grounds are arbitrary and could have substantial consequences with respect to competition in space between fishing and other uses. Different thresholds for inclusion in HIFA need to be considered when moving to policy and management applications. In addition, the clustering of HIFA, as in any hierarchical approach, has to be considered as an exploratory tool: the clusters may change over time following the seasonal changes in patterns of fishing activity.

## Conclusions

In summary, our study showed how the availability of detailed fishing activity data gives new insights into the spatial dimension of the socio-economic interactions between coastal communities and HIFA. These explicit spatial relations of the fishing activity can enrich the classical fisheries bioeconomic models which, given the relatively high level of aggregation of the available fishing activity data, can only look at the relation between fleets and fish stocks without considering the local geography. In addition, the open nature of AIS data offer an accessible data source to conduct such analyses, even at a very large scale, complementing more traditional and national administration based sources for fishing activity data which are not assembled across the EU at the level of primary data. The limitations of the AIS and EUMOFA datasets have partially affected the results of the current study but the methodology provides a novel framework for the analysis of the effects of fishing in spatially explicit terms and accounting for socio economic covariates.

## Supporting information

S1 Data (ZIP)
